# SARS CoV-2 IgG seropositivity post-vaccination among dental professionals: a prospective study

**DOI:** 10.1186/s12879-023-08534-z

**Published:** 2023-08-18

**Authors:** Irena Duś-Ilnicka, Marta Mazur, Anna Rybińska, Małgorzata Radwan-Oczko, Kamil Jurczyszyn, Anna Paradowska-Stolarz

**Affiliations:** 1 Oral Pathology Department, Wroclaw Medical University, ul. Krakowska 26, Wrocław, 52-425 Poland; 2 Department of Oral and Maxillofacial Sciences, Sapienza University of Rome, Rome, 00161 Italy; 3 Department of Oral Surgery, Wroclaw Medical University, Krakowska 26, Wrocław, 50-425 Poland; 4 Division of Dentofacial Anomalies, Department of Orthodontics and Dentofacial Orhopedics, Wroclaw Medical University, Krakowska 26, Wrocław, 52-425 Poland

**Keywords:** SARS-CoV-2, IgG, IgG count, COVID-19 vaccination, Vaccination, Immunity, Dental professionals

## Abstract

**Background:**

The COVID-19 pandemic has spread very rapidly around the world. Various regional and national lockdowns were imposed to control the spread. Meanwhile, vaccine development and population vaccination were the next steps for pandemic control. Workers in the dental field, both dentists and dental assistants, however, were close to the sources of aerosol generated during dental procedures and thus were the group of workers the most exposed to COVID-19 infection. The aim of our study was to monitor the immune response before and after the vaccine in a high-risk population, composed by dental professionals.

**Methods:**

A clinical prospective study was carried out among dental professionals at the Academic Dental Polyclinic, Wroclaw Medical University (Wrocław, Lower Silesia region, Poland). Blood samples were collected at an interval of one year – March/April 2020, before the vaccination against COVID-19, and April 2021, after the vaccination. The analysis was performed on serum with four different methods: qualitative, semi-quantitative, and quantitative IgG count for SARS-CoV-2, and SARS-CoV-2 neutralizing antibodies.

**Results:**

A total of 42 healthy adult volunteers participated in the study. The results showed a statistically significant difference (p < 0.05) in antibody levels before and after vaccination (1st and 2nd measurement) for each test method. The tests that were used affected the results and the test that showed the strongest relationship with the result was the Qualitative test.

**Conclusions:**

Dental professionals are the adult working population most at risk for COVID-19. Monitoring SARS-CoV-2-status-related seropositivity can provide useful information occupational risk factors for dental professionals.

## Introduction

SARS-CoV-2 (severe acute respiratory syndrome coronavirus-2) is an RNA virus that causes COVID-19 disease, whose spreading led World Health Organization (WHO) to classify this disease as a pandemic in March 2020 [1]. The risk of spreading the disease is considered high, especially among dental services, which is directly connected to the production of aerosols in the dental office [2, 3]. Dental offices were also the primary places to search for the oral symptoms of vaccination against COVID-19, and COVID-19 disease [4, 5].

The main factor fighting this virus is antibody production [6]. Although many diagnostic methods help in detecting SARS-CoV-2 RNA, in particular,l real-time RT-PCR technique, the detection of the immunoglobulins would be more adequate to assess seropositivity and provide an insight into the asymptomatic disease progression [7]. IgM and IgG antibodies play an essential role in epidemiological studies, as used for the algorithm recommended by the Centers for Disease Control and Prevention (CDC) [7].

Even though it is commonly used for diagnostic purposes, Point Of Care Testing (POCT) for IgM and IgG is not, according to the FDA and CDC guidelines, a way to detect infections [7]. This applies to all types of antibodies (e.g. IgG, IgM, IgA). However, those types of tests were used as the most popular and accessible way for diagnosing, especially in the first days of the pandemic. The most popular type of antibody test used during the pandemic for POCT is the LFIA (lateral flow immunochromatography assay). The result of this test is visualized as a vertical line, visible to the naked eye. Therefore, the test does not need the highly advanced skills of laboratory personnel. However, these tests have variable specificity and sensitivity (96.6–99.7% and 49.3–79.3%, respectively) [8, 9]. This technology allows for the detection of viral antigens, but also IgG and IgM antibodies. The other type of test that allows for the detection of SARS-CoV-2 antibodies is the chemiluminescence assay (CLIA). In this case, a luminophore marker is used. According to the producer of Shenzhen YHLO Biotech kit, the specificity of the test is 92.2% in the case of IgM and 100% in IgG at a sensitivity of 73.3% and 76.7%, respectively [10]. A very popular laboratory test for the detection of antibodies is ELISA (enzyme-linked immunosorbent assay). The anti-SARS-CoV-2 antibodies in serum or plasma are bound to the plates coated with SARS-CoV-2 antigens [11, 12].

One of the most specific ways of diagnosing the immunological response presented upon actual contact with the virus is to perform a measurement of neutralizing antibodies. One of the breakthroughs for laboratory medicine was the use of the S1 and S2 subunit components of SARS-CoV-2 spike protein in serological diagnostics. Those subunits are located on the outer envelope of the virion. The S1 subunit plays a critical role in the viral attack on the endothelial cells of infected individuals. It binds to the ACE2 cell receptor, which causes viral fusion with the cell membrane and entry into the cell. The anti-S antibodies formed during SARS-CoV-2 infection, the so-called neutralizing antibodies, reduce the virulence of the virus by blocking the SARS-CoV-2 spike RBD (receptor-binding domain) preventing the binding with the ACE2 receptor. To sum up, the presence of neutralizing antibodies prevents fusion with the cell membrane and further viral entry and infection [12–14].

Although serological testing seems to be a good diagnostic method, it has some limitations. Because seroconversion can take up to three weeks after the infection, it does not truly reveal if the individual is currently infected. In the case of SARS-CoV-2, the majority of the individuals develop seroconversion between seven and eleven days after exposure [15, 16].

Human saliva contains viral particles, and dental handpieces and ultrasonic devices produce aerosols whose droplets may fall around and contaminate both the air and surfaces [17–21]. Due to those factors, dentistry is thought to be one of the most hazardous professions when SARS-CoV-2 infection is taken into account [20]. According to a Colombian research study by Plaza-Ruíz et al. [21], 96% of dentists were afraid that COVID-19 infection was a risk for them. Therefore, they considered reducing working hours (circa 80% of the participants in the study) or changing their profession (18.15%). Considering that dentists are more prone to SARS-CoV-2 infection, protection tools and disinfection agents are used in the dental office to fight the contamination of the surroundings and the equipment [22].

The article intends to compare four methods of diagnostics for SARS-CoV-2 IgG seropositivity after vaccination among a group of high exposure – dental professionals. The primary aim of the present study was to evaluate the seropositivity from the blood of dental personnel (dentists and dental assistants) working during the COVID-19 pandemic. The secondary aim was to evaluate the suitability of different types of tests to perform the analysis of individuals’ SARS-CoV-2 IgG count, and the evolution of laboratory testing for seropositivity against it during the pandemic.

## Materials and methods

The protocol was approved by the Wroclaw Medical University Bioethical Committee (approval number 576/2020) and informed consent was obtained from all individuals. All the procedures were in accordance with the 2013 Helsinki Declaration and its later amendments or comparable ethical standards [23]. The analysis was performed on biobanked biosamples of serum with the use of four different methods: qualitative, semi-quantitative, and quantitative IgG count for SARS-CoV-2, and SARS-CoV-2 neutralizing antibodies. In order to support the secondary aim of the study, the measurements on each sample (patient) were performed with the use of each of the four methods, resulting in the possibility of comparing them internally.

### Study design and setting

A prospective clinical study was carried out at the Academic Dental Polyclinic, Wroclaw Medical University (Wrocław, Lower Silesia Region, Poland).

The inclusion criteria were as follows:

i) Dental workers (dental assistants) actively working in dental studios from the first wave of the pandemic, and did not withdraw from work for longer than two weeks. Dental assistants working full time (7 h 30 min each day).
ii) Volunteers without any untreated chronic disease.
iii) Absence of any pulmonary infections with probable COVID-19 background.

**Fig. 1:**
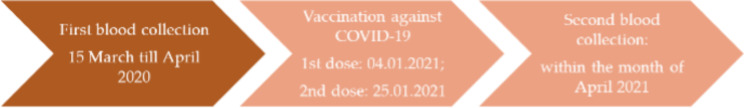
Description of the study protocol and dates

Blood samples were obtained at an interval of one year – March/April 2020, before the vaccination against COVID-19, and April 2021, after the vaccination. (Fig. 1)

### Venous blood collection and safety procedures

Venous blood was collected according to good laboratory practice in accordance with the safety procedures for COVID-19 described in the details of the previous research by the authors [3].

### COVID-19 (SARS-CoV-2) IgG tests

#### COVID-19 (SARS-CoV-2) qualitative IgG ELISA test

First, all clinical samples of serum were analyzed for the qualitative determination of specific IgG class antibodies against SARS-CoV-2 in blood serum using a commercial IVD-certified enzyme-linked immunosorbent assay (ELISA) kit (COVID-19 (SARS-CoV-2) IgG ELISA, Demeditec Diagnostics GmbH, Germany, Lot. COVG-009). As provided by the manufacturer, microtiter plates used in the ELISA technique are coated with specific antigens to bind the corresponding antibodies of the sample. The manufacturer of this test does not specify which proteins the microtiter plates are covered with but dedicates them to verify antibodies produced during natural infection through contact with the antigens of the pathogen or potentially after vaccination (although the COVID-19 vaccine was unknown at the time the first version of the test was designed).

The test was carried out according to the procedure recommended by the manufacturer. Test samples were diluted 100X and then spotted into specific wells of microtiter plates. After incubation and washing, the wells from unbound sample material, a horseradish peroxidase (HRP) labelled conjugate was added to bind to the captured antibodies. In the second washing step, the unbound conjugate was removed. The immune complex formed by the bound conjugate was visualized by adding Tetramethylbenzidine (TMB) substrate which resulted in a blue reaction product. The intensity of this product was proportional to the amount of specific antibodies in the sample. Sulphuric acid was added to stop the reaction. This produced a yellow endpoint color. Final absorbance values for each standard/control and sample in the plate layout were taken at 450 nm with a correction absorbance at 620 nm using ELISA spectrophotometry (EPOCH).

In accordance with the producer’s manual, samples with a concentration of < 9 U/ml were considered non-reactive, those ranging 9–11 U/ml were considered equivocal, and samples > 11 U/ml were considered reactive. In the case of equivocal sample results, it was recommended by the manufacturer to repeat the test with a fresh sample in two to four weeks [24]. The manufacturer provided a set of three calibrators and three levels of controls.

Reagents and buffers were prepared according to the manufacturer’s instructions. For the evaluation of the assay, it is a precondition that the absorbance values of the blank should be below 0.100; the absorbance values of the negative control should be below 0.200 and should be smaller than the cut-off; the absorbance values of the positive control should be greater than the cut-off; and the absorbance values of the cut-off control should be within the limits of 0.150–1.300. The results of the level of IgG in Units [U] were arrived at by mathematical testing using the formula provided in the test insert.

#### COVID-19 (SARS-CoV-2) quantitative IgG ELISA test

Then, the level of specific IgG-class antibodies against spike protein of SARS-CoV-2 in blood serum was determined using an IVD-certified enzyme-linked immunosorbent assay (ELISA) kit (COVID-19 (SARS-CoV-2) quantitative IgG ELISA, Demeditec Diagnostics GmbH, Germany, Lot. DECOV1901Q) according to the manufacturer’s instructions [24].

As provided by the manufacturer, the microtiter plates used in the ELISA technique are coated with specific antigens such as trimeric spike protein (S) to bind proper antibodies in the sample.

According to the manufacturer’s recommendations, the set is dedicated to diagnosing patients suspected of COVID-19 disease or asymptomatic SARS-CoV-2 infection and monitoring antibody levels during/after COVID-19 disease, and before/after COVID-19 vaccinations.

This test was carried out analogous to the qualitative test performed earlier with the difference that in the quantitative assay procedure, the manufacturer provided a set of five calibrators CAL 1–5. In this test, the calibration samples were performed in duplicate. In addition, each test was verified using two levels of controls attached to the kit. For the evaluation of the assay, it is a precondition that the absorbance values of the blank should be below 0.150; the absorbance values of the first calibration should be greater than 1 and greater than the absorbance values of the second calibrator. The absorbance values of the second calibrator should be greater than the third, of the third than the fourth, of the fourth than the fifth. The obtained absorbance values of the blank, controls 1 and 2, and calibrators 1–5 were within the ranges specified in the Quality Control certificate.

Again, test samples were diluted and then spotted into defined wells of microtiter plates. After incubation and washing, the wells from unbound sample material, a horseradish peroxidase (HRP) labeled conjugate was added to bind to the captured antibodies. In the second washing step, the unbound conjugate was removed. The immune complex formed by the bound conjugate was visualized by adding Tetramethylbenzidine (TMB) substrate which resulted in a blue reaction product. The intensity of this product was proportional to the amount of specific antibodies in the sample. Sulphuric acid was added to stop the reaction, producing a yellow endpoint color. Final absorbance values for each standard/control and sample in the plate layout were taken at 450 nm with a correction absorbance at 620 nm using ELISA spectrophotometry (EPOCH).

The kit provided calibrators from the range of 0 to 200 AU/ml. A graph was made based on the obtained data, in which the mean absorbance values of the calibrators are marked on the y-axis and the nominal concentration of calibrators on the x-axis. Based on this curve, the concentration of antibodies in the serum samples collected from the subjects before and after vaccination was determined. The results of the level of IgG are provided in AU/ml.

The concentration values of IgG against SARS-CoV-2 of the positive control should be within the indicated limits of 48–126,73 AU/ml, and the value of the negative control should be within the limits of 0–10 AU/ml.

#### SARS-CoV-2 neutralizing antibody detection ELISA kit

Finally, all clinical samples of serum were analyzed using Cayman Chemical Company’s SARS-CoV-2 Neutralizing Antibody Detection ELISA Kit for the quantitative measurement of neutralizing antibodies against SARS-CoV-2 in human plasma.

Cayman’s ELISA Kit provided a robust and easy-to-use platform for identifying the neutralizing antibodies of the SARS-CoV-2 spike S1 receptor-binding domain (RBD) and angiotensin-converting enzyme 2 (ACE2) interaction, produced during SARS-CoV-2 infection.

In the performed test, the level of SARS-CoV-2 spike protein-specific neutralizing antibodies was quantified in order to check the history of infection and determine the response to vaccination against Covid-19.

The assay uses a recombinant rabbit Fc-tagged SARS-CoV-2 spike S1 RBD that binds to a plate pre-coated with an anti-rabbit Fc-specific antibody. A recombinant His-tagged ACE2 protein binds the SARS-CoV-2 spike S1 RBD and the complex is detected with an HRP-conjugated anti-His antibody.

Final absorbance values for each blank/standard/control and sample in the plate layout were taken at 450 nm using ELISA spectrophotometry (EPOCH). Samples, standards, reagents, and buffers were prepared according to the manufacturer’s instructions.

A curve was generated from the obtained data, in which the mean absorbance values of the calibrators are marked on the y-axis and the corresponding nominal concentration on the x-axis.

The kit provided calibrators from the range of 7.81 to 1,000 ng/ml. On the basis of this curve, the concentration of neutralizing antibodies in the serum samples collected from the subjects before and after vaccination was determined. The results of the antibody level are provided in ng/ml. In the case of results < 3000 ng/ml, the diagnostic test was considered negative.

### Statistical analysis

“Statistica” software version 13.3 (StatSoft, Kraków, Poland) was used to calculate all statistical tests. The statistically significant level was set as 0.05. The normality of distribution was confirmed with the use of the Shapiro–Wilk test. Due to the lack of normal distribution, a nonparametric sign test was applied to compare the concentrations of antibodies between two measurements. Pearson Chi-square test was used to estimate the dependence between the applied test and its results. The power of test for each examined group was calculated based on the number of samples, means, and standard deviation. The power of the test for each group was calculated based on the differences between means and the number of samples.

For the purposes of the statistical analysis, the Null hypotheses were stated as follows:

#### Hypothesis 1

There are no differences between the level of antibodies measured before and after vaccination.

#### Hypothesis 2

Results of each test do not depend on the test methods.

## Results

A total of 42 subjects were enrolled with an age range of 25–50. All the subjects were vaccinated during the same time period. The characteristics of the enrolled subjects are shown in Table 1.

**Table 1:** Study group presentation at the moment of blood collection

	Number of personnel and percentage of the study group
Women	34 (81%)
Men	8 (19%)
Chairside assistants	17 (40.5%)
Dental medical doctors	25 (59.5%)
Vaccinated with Comirnaty, Pfizer at the moment of the 2nd blood collection	38 (90.1%)
Vaccinated with COVID-19 Vaccine AstraZeneca at the moment of the 2nd blood collection	4 (9.9%)

In the case of neutralizing antibodies, the research was performed on 27 subjects in the timeframe of one year. Table 2 shows the results of the sign test comparing the levels of antibodies before and after vaccination using each test described in the Methods section. In all the cases, the results revealed statistical differences between the first and second measurements. It is important to underline that in the case of Qualitative diagnostic tests used, the p-value equals only 0.03 which is close to statistical significance, but due to the power of the test at the level of 13%, the authors are not able to reject the null hypothesis.

In the cases of missing results where the number of individuals differed from the previously set number of 42 volunteers, the reason was the limited volume of the serum that was insufficient to perform the analysis. This has been reflected in the number of individuals in Table 2 in the “N” column.

**Table 2:** Results of the sign test. Comparison of antibodies concentration between two measurements (I – first measurement, II – second measurement, SD – standard deviation, N – number of samples, p – probability value)

	Unit	Median	Mean	SD	Difference	Difference SD	N	p value	Power of test
Qualitative (1st BV)	U	5.98	6.38	2.47	-0.74	1.57	21	*0.030000*	13.35%
Qualitative (2nd AV)	U	7.18	7.11	3.15					
Semi-quantitative (1st BV)	AU/ml	0.80	2.82	6.79	-45.79	17.74	40	*0.000000*	100.00%
Semi-quantitative (2nd AV)	AU/ml	55.84	48.61	17.49					
Quantitative (1st BV)	AU/ml	0.00	6.84	26.04	-208.93	81.74	38	*0.000000*	100.00%
Quantitative (2nd AV)	AU/ml	256.00	215.77	81.13					
Neutralizing antibody (1st BV)	ng/ml	2200.00	1952.17	1515.34	-43859.35	72121.46	23	*0.000175*	79.59%
Neutralizing antibody (2nd AV)	ng/ml	22500.00	45811.52	72262.94					

1st BV: first measurement before vaccination, 2nd AV: second measurement after 2nd dose of the vaccination

**Fig. 2:**
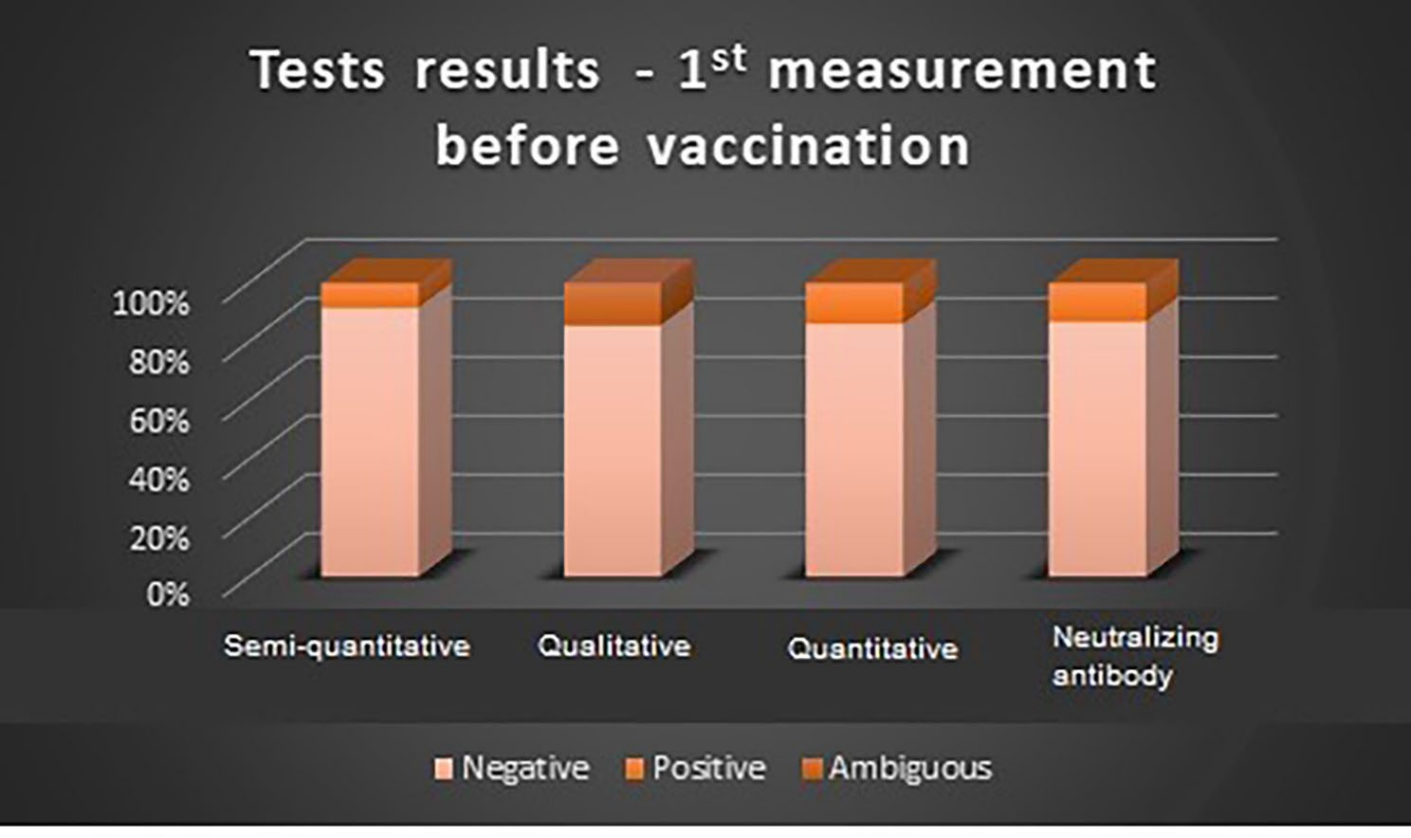
Results of each IgG SARS-CoV-2 test during the first measurement before vaccination

**Fig. 3:**
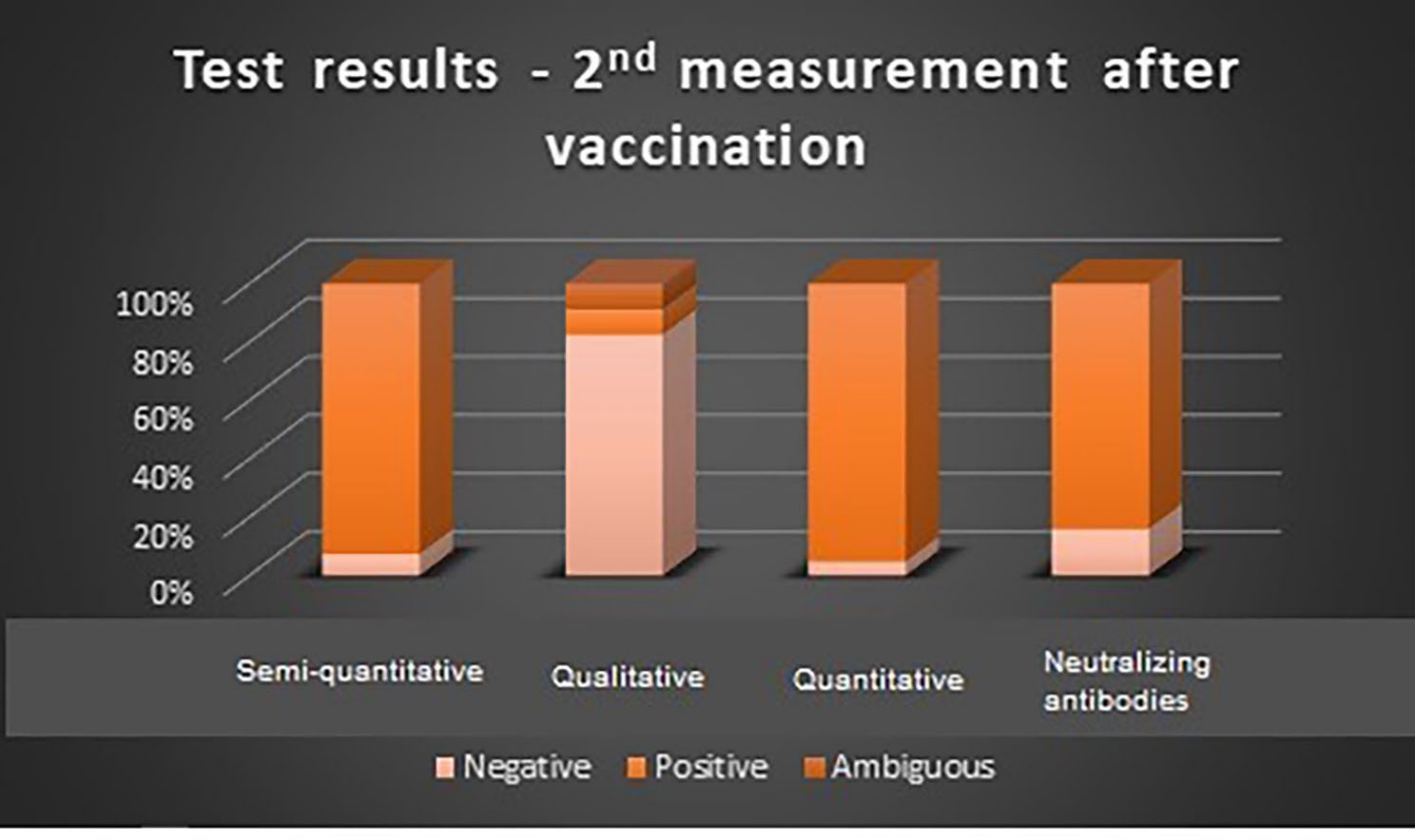
Results of each IgG SARS-CoV-2 test during the second measurement

In the first measurement before vaccination, the highest value of negative results was seen for the semi-quantitative test (96.67%). Ambiguous results were reported only in the case of qualitative tests, in this group the lowest value of negative test was noted (85.71%). In the second measurement after vaccination, the lowest positive results were observed in the case of quantitative tests (4.88%). Similar to the first measurement, ambiguous results were seen only in the case of qualitative tests at the level of 8.70%. Test results before and after vaccination are shown in Figs. 2 and 3.

In the first and second measurements, (p < 0.05), the result depends on the specific test. At the first measurement, the results were below but close to the significance level, since p = 0.01, however, it was not reached. The reason might probably be setting the “ambiguous” part of the results for this test, which were not set in other tests (semi-quantitative, quantitative, and neutralizing antibodies tests). The higher the value of the Chi-square test, the stronger the relationship between the examined properties. In this case, the Chi-square value was 16.74 in the first measurement before vaccination in contrast to 76.80 in the case of the second measurement after the second dose of vaccination. The relationship between the type of test and its result is approximately 4.6 times stronger in the group after vaccination than in the group before vaccination (Table 3).

**Table 3:** Results of the Chi-square test. Estimation of dependency between type of test and its result. (Chi^2 – Chi-square test value, df – degree of freedom, p – probability value)

	1st BV			2nd AV		
	Chi^2	df	p	Chi^2	df	p
Pearson Chi-square	16.74242	df = 6	*p = 0.01028*	75.79086	df = 6	*p = 0.00000*

Legend: 1st BV: first measurement before vaccination, 2nd AV: second measurement 1 month after 2nd dose of the vaccination

If p > 0.05, the data were assumed to be not related to each other, i.e., the test result does not depend on the selected method. In Qualitative IgG testing when compared with other methods, p was always less than 0.05, i.e., the test result depended on the test method (especially during the second measurement after the vaccination process). At the first measurement before the vaccination process, p was lower than 0.05. This may imply that tests perform better in evaluating scores of 0 than 1 (Table 4).

In the remaining pairs, at the first measurement, p was equal to or higher than 0.054, so it was assumed that there was no relationship between the method and the test result.

**Table 4:** Results of Chi-square test. Estimation of dependency between the type of test and its result for pairs. (Chi^2 – Chi-square test value, df – degree of freedom, p – probability value, x – the same pair)

1st measurement (before the vaccination)												
	Semi-quantitative			Qualitative			Quantitative			Neutralizing antibody		
	Chi^2	df	p	Chi^2	df	p	Chi^2	df	p	Chi^2	df	p
Semi-quantitative	x	x	x	6.95	2	0.031	0.56	2	0.755	0.341	2	0.843
Qualitative	6.95	2	*0.031*	x	x	x	8.06	2	*0.018*	6.027	2	*0.049*
Quantitative	0.56	2	0.755		2	0.018	x	x	x	0.009	2	0.996
Neutralizing antibody	0.34	2	0.843	6.03	2	0.049	0.01	2	0.996	X	x	x
**2nd measurement (after the vaccination)**												
	Semi-quantitative			Qualitative			Quantitative			Neutralizing antibody		
	Chi^2	df	P	Chi^2	df	p	Chi^2	df	P	Chi^2	df	p
Semi-quantitative	x	x	x	44.49	2	*0.000*	0.21	2	0.899	1.23	2	0.539
Qualitative	44.49	2	*0.000*	x	x	x	47.88	2	*0.000*	27.44	2	*0.000*
Quantitative	0.21	2	0.899	47.88	2	*0.000*	x	x	x	2.32	2	0.313
Neutralizing antibody	1.23	2	0.539	27.44	2	*0.000*	2.32	2	0.313	x	x	x

## Discussion

Biobanked samples collected from dental workers through the pandemic waves represent a unique material to test the immunological response to the vaccine and also enable an analysis of seropositivity during the pandemic [3]. Because of the risk that dental workers are facing during the pandemic regarding exposure to oral bioaerosol, the question asked by the research team within this paper was if the initial work in harmful environments has brought the risk of infections to dental personnel without their awareness. As per the quantitative diagnostic of SARS-CoV-2 IgG antibodies, out of the total of 42 dental workers, four (9.52%) tested positive before the vaccination in April 2020. In comparison with a cross-sectional study from the UK, seroprevalence among dental workers was reported at 16.3% [25]. This higher result might be caused by the researchers using an assay simultaneously measuring three types of antibodies: IgG, IgA, and IgM directed against the spike glycoprotein of SARS-CoV-2. This might have facilitated the detection of antibody response against the antigen and resulted in a higher percentage than that revealed in the current study [25]. In a study conducted by Ribeiro et al., on the other hand, 6.5% of the dentists were IgG-positive [26]. Similar study was conducted by Shields et al. however the followup period provided by the researchers in that study was longer [25]. Manuscripts of topics refering to the dental professionals working in the bioaerosol environment, can additionally present the iportance of strenghtening the occupational risks, as stated also by Shields et al. [25].

When referring to the Qualitative method of testing, only one of the four positively diagnosed volunteers received the same positive result, which has left three of them diagnosed with insufficient specificity (2.38% of the volunteers testing positive in Qualitative SARS-CoV-2 IgG diagnostics). The specificity of the different types of IgG SARS CoV-2 tests in comparison with the neutralizing antibodies was discussed for smaller [27] and larger sample sizes [28] by the research audience, but evidence is lacking in the samples from professionals working directly with oral bioaerosol. This is especially true for prospective studies that would involve dental professionals prior to the vaccination, as this process was started in Poland in December 2020, which provided our research team with a window of 10 months to collect the samples – from 11 to 2020 when the pandemic had been declared by WHO. However, lower sensitivity and specificity of the IgM in comparison to IgG has been reported [12], it has also been suggested that the highest overall sensitivity can be achieved from an IgM-IgG combined assay compared to nucleic acid testing and single diagnostics of IgM and IgG [25, 29]. This study, however, did not aim to diagnose the disease, but to evaluate if dental workers were affected by the disease without their knowledge, and for this purpose, IgG count was considered the most reliable method.

Multiple research studies have shown that the main line of defense against the SARS-CoV-2 virus, especially in primary infection, is the production of antibodies [6]. As per seropositivity after the vaccination, it is important to provide diagnostics after the complete cycle of vaccination. The possible differences in the levels of IgG count between different types of vaccines are underlined when the antibody levels are diagnosed after single shots of vaccinations [30]. This parameter has been excluded by setting the diagnostics after a finished procedure of vaccination. The present study did not focus on measuring IgG levels in individuals after receiving a booster.

Throughout the pandemic, the laboratory diagnostic has passed through waves of changes in biohazard handling, where the antigen testing on lateral flow tests previously performed on a bench has started to be done under a BSL-2 hood, for the safety of the operator. Salivary and sputum samples collected at the bedside before the pandemic without specific biohazard handling have begun to pose a significant risk to the operators’ health. That is why guidelines had to be introduced that would strictly define how to proceed with the changing laboratory needs and settings [31, 32]. The evolution of laboratory methods has concerned not only biohazard handling but also the implementation of serologic tests to the market due to urgency, which usually has resulted in limited validation by the developer [33]. For this reason, a comparative assessment was provided for different methods of IgG diagnostics in dental workers. The test that showed the strongest relationship with the result was the Qualitative IgG test, developed at the beginning of the first wave of COVID-19. This is understandable, and further confirmation of the high concordance between results obtained with the use of quantitative tests would provide a picture of how the tests evolved in time during the pandemic of COVID-19.

### Limitations

The present study was conducted on a considerably small number of individuals who were similar in terms of health status, the practiced profession, proximity to a likely source of COVID-19 infection, i.e. the setting of a dental practice, and timing of blood draws and COVID-19 vaccinations. Certainly, future studies could look at a larger sample of dental practitioners and monitor the status of possible COVID-19 re-infection following vaccine boosters over the long term by analyzing immune responses, as this results represent the good practice also for the analysis of occupational risk factors.

## Conclusions

1. A statistical difference in antibody levels before and after vaccination (1st and 2nd measurement) for each test method was observed (therefore Null Hypothesis 1 is rejected), except for the first – Qualitative test, where the test power was low and the p-value at borderline significance.
2. The test method used affected the result. The test that showed the strongest relationship with the result was Qualitative IgG (therefore Null Hypothesis 2 is rejected).

## Data Availability

Data are available upon a reasonable request from the corresponding author.
